# Bushen Huoxue Formula Modulates Autophagic Flux and Inhibits Apoptosis to Protect Nucleus Pulposus Cells by Restoring the AMPK/SIRT1 Pathway

**DOI:** 10.1155/2022/8929448

**Published:** 2022-05-27

**Authors:** Shang Gao, Nianhu Li, Renchang Chen, Youxiang Su, Yun Song, Songlin Liang

**Affiliations:** ^1^First Clinical Medical College of Shandong University of Traditional Chinese Medicine, Jinan, China; ^2^Affiliated Hospital of Shandong University of Traditional Chinese Medicine, Jinan, China

## Abstract

**Background:**

Low back pain (LBP) has the characteristics of chronic and persistence, which is a heavy social burden. Intervertebral disc degeneration (IVDD) is a major cause of LBP. The typical features of IVDD are extracellular matrix (ECM) degradation and nucleus pulposus cell (NP) apoptosis. Bushen Huoxue Formula (BSHXF) has good clinical effects on LBP. However, the mechanism of BSHXF affecting ECM and NP cells is still unclear. *Aim of the Study*. In this study, the impact of BSHXF on autophagy and apoptosis of NP cells was studied through the AMPK/SIRT1 pathway. *Material and Methods*. NP cells were extracted through the digestion of collagenase and trypsin, and the components of BSHXF were identified. Cell Counting Kit-8 was applied to detect the impact of BSHXF on NP cells. Mitochondrial function was detected using MitoTracker assay, ATP kit, and SOD kit. Autophagy and apoptosis were detected by RT-qPCR, western blotting, and flow cytometry.

**Results:**

BSHXF promoted NP cell survival in a concentration-dependent manner, and the elimination of rat serum did not increase cell proliferation; TNF-*α* accelerated ECM degradation, ROS accumulation, and NP cell apoptosis and decreased autophagic flux. BSHXF restored mitochondrial function and autophagic flux. In addition, AMPK/SIRT1 pathway activation was associated with IVDD.

**Conclusions:**

BSHXF regulates autophagy and enhances autophagic flux to suppress excessive ROS production and restore mitochondrial function in an AMPK/SIRT1-dependent manner. However, the protection of BSHXF on TNF-*α*-treated cells was eliminated by 3-MA. Furthermore, the protective impact of BSHXF on ECM degradation and apoptosis induced by TNF-*α* was restrained by an AMPK inhibitor. Therefore, maintaining the proper autophagy illustrates treatment strategy for IVDD.

## 1. Introduction

Degeneration of the intervertebral disc (IVD) is the major cause of low back pain (LBP), which not only affects the quality of life of patients but also dramatically increases the economic burden on society [1–3]. LBP is a complex process whose mechanism and treatment strategy need to be further refined. IVD plays a key role in spinal diseases, mainly occurring at the cellular and molecular levels, resulting in changes in the tissue homeostasis and physiology of the nucleus pulposus (NP), leading to a decline in the ability to resist stress [4]. IVD is constituted by three main parts: nucleus pulposus (NP) in the center, annulus fibrosus (AF) of the periphery, and cartilage endplate (CEP), which interact to ensure the disc homeostasis. The stability of each structure of the IVD ensures that IVD cells respond to biological and chemical regulation in response to changes in signaling environment ([1, 5, 6]). Physiological function and normal structure of IVD need to preserve under the action of NP cells, which secretes two main components, aggrecan and type II collagenase [7]. A great many evidences now manifest that abnormal NP cell functions, including apoptosis, ECM degradation, and cytokine oversecretion, are key to IVDD pathogenesis [8]. The risk factors for intervertebral disc degeneration (IVDD) include oxidative damage, genetic factors, local instability, diabetes, and mechanical load [4, 9, 10]. The main characteristics of IVD are decreased ECM, increased reactive oxygen species (ROS) caused by oxidative damage, and the increases in various inflammatory factors [11–15]. Two cytokines, interleukin-6 (IL-6) and tumor necrosis factor-*α* (TNF-*α*), engage in the development of IVDD as major inflammatory factors [16]. TNF-*α* possesses pleiotropic proinflammatory activity that enables to cause inflammation, nerve pain, and cell apoptosis. Therefore, TNF-*α* is well-known for its cytotoxic effect leading to inflammatory reaction and apoptosis in NP cells [8, 12, 17]. IVDD, mechanical injury, and cytokine induction, which may cause mitochondrial damage. Mitochondrial dysfunction may increase the excessive production of ROS, resulting in excessive inflammation, metabolic disorder, and increased apoptosis [18–20]. Mitochondria can produce harmful ROS through oxidative phosphorylation defects. ROS can induce oxidative stress in cells, which in turn damages mitochondrial function, and the impaired mitochondria are effectively removed through a selective autophagy called autophagy. Two different processes, autophagy and apoptosis, play an important part in IVDD [21–23]. Obviously, apoptosis and autophagy are closely related but mutually exclusive cellular processes, which play an indispensable part in the mechanism of IVDD [24–26].

Autophagy, which is reflected in both physiological and pathological processes, is the process of engulfing damaged proteins or organelles, encapsulating them into vesicles, fusing with lysosomes to form autophagic lysosomes, and degrading their encapsulated contents [27–29]. The dynamic process of fusion of autophagosome and lysosome, degradation and regeneration of damaged cells, can be summarized by the autophagic flux [30–32]. Autophagy dysfunction can be manifested in the following aspects, inhibiting autophagy formation, blocking autophagic lysosomal fusion, and destroying lysosomal function [33–36]. It was reported that degenerative diseases, such as osteoarthritis, intervertebral disc degeneration, neurodegenerative diseases, and age-related macular degeneration, are in connection with senescence and cell apoptosis [18, 37]. In recent years, studies have demonstrated that oxidative stress gives rise to autophagy dysfunction using rat NP cells [38]. Therefore, upregulation of autophagy flux may be an active therapeutic strategy for IVDD. ECM metabolism is primarily regulated by two catabolic enzyme systems, including matrix metalloproteinases and metalloproteinases, and proved to have an intimate connection with the development of degenerative cartilage diseases [28, 39, 40]. The level of MMP1, 3, and 9 is highly expressed in the degenerative NP. It was reported that the expression of aggrecans ADAMTS 4, 5, and 15 significantly increased in degraded tissues compared with normal tissues. In addition, Il-1, IL-6, TNF-*α*, and other inflammatory factors also play an important part in IVDD. It was shown that the proinflammatory factor IL-1 is the most important cytokine in the release of a variety of proinflammatory mediators, including TNF-*α*, IL-6, and MMPs. The inflammatory factor disrupt the extracellular matrix and cause intervertebral disc tissue damage [41].

When the cells are in a state of energy deficiency, such as starvation and motor physiological process, AMPK is activated. Once activated, AMPK regulates ATP levels both by inhibiting ATP consumption and by activating ATP production [42, 43]. AMPK plays an important role in cellular energy homeostasis and is regulated and controlled by a variety of upstream signals. Various signaling regulators can be regulated according to the state of nutrients, energy, and metabolites in the cellular environment. In addition, AMPK signaling pathway is involved in cell energy maintenance, nutrient supply, and regulation of autophagy and apoptosis. Sirtuin, with highly conserved NAD^+^ binding domain and catalytic functional domain, is a highly conserved deacetylase from bacteria to humans, which can regulate the acetylation modification and ADP ribosomal modification of a variety of proteins [44, 45]. Sirtuins occupy an indispensable position in all sorts of cellular physiological and pathological processes including inflammation, aging, oxidative stress, energy metabolism, and autophagy [46]. These proteins are closely associated with degenerative diseases related to cellular aging such as diabetes, neurodegenerative diseases, and cardiovascular diseases [30, 47, 48].

Traditional Chinese medicine (TCM) has been applied in many countries for thousands of years, especially China. Compared with surgical treatment of intervertebral disc degeneration, TCM therapy has the advantages of good efficacy, low cost, and small side effects and is more safe and effective [49, 50]. In the theory of TCM, the mechanism of IVDD belongs to kidney deficiency and blood stasis. The corresponding treatment methods are tonifying kidney and warming yang, promoting blood circulation, and removing blood stasis. Bushen Huoxue Formula (BSHXF) is the representative prescription of tonifying kidney and activating blood [51, 52]. The BSHXF consists of five herbs, among which Rehmanniae Radix can tonify the kidney and promote blood circulation [53], and Salvia miltiorrhiza can promote blood circulation and remove blood stasis [54]. This prescription plays the role of treating different diseases with the same treatment, that is, tonifying kidney and activating blood to treat kidney deficiency and blood stasis syndrome.

We investigated the impact of BSHXF on NP cells in this study. The impact of BSHXF on autophagic flux and the inhibition of apoptosis, TNF-*α*-induced inflammatory responses, ECM degradation, and mitochondrial function under oxidative stress were examined. The mechanism of BSHXF in treating IVD and fighting against inflammatory response and oxidative stress by activating AMPK/SIRT1 signaling pathway needs further study.

## 2. Material and Methods

### 2.1. IVD Sample and Ethics Statement

Lumbar intervertebral disc tissue was gained from 12 patients (6 males and 6 females; aged 25 to 60 years) undergoing deformity correction surgery to treat idiopathic scoliosis. This research proposal was authorized by the Ethics Committee of the Affiliated Hospital of Shandong University of Traditional Chinese Medicine (2021 Ethics NO.008—KY).

### 2.2. Chinese Herbs and Production of BSHXF

BSHXF (Table 1), which is composed of five kinds of herbal medicine (Aconitum carmichaelii Debx, Rehmannia glutinosa Libosch, Morinda officinalis, Salvia miltiorrhiza Bunge, and Curculigo orchioides Gaertn), was obtained from the Affiliated Hospital of Shandong University of Traditional Chinese Medicine (Shandong, China). All herbs were authenticated by experts from the School of Pharmacy of Shandong University of Traditional Chinese Medicine. The herbs in BSHXF were mixed and soaked for 30 minutes and decocted for 40 minutes. The process was carried out twice. The first and second decoctions were collected and concentrated to 1 g/ml. The filtered liquid was stored at -4°C.

### 2.3. Identification of Herbal Materials

We combined the prepared BSHXF solution with the mixture containing 0.3 mg/ml L-2-chlorophenylalanine methanol. The mixed solution was centrifuged at 300 × *g* at 4**°**C. Then, we took out appropriate amount of supernate with a pipette, filtered through a filter, and stored at phial. The sample was analyzed by an analysis system composed of a liquid-mass coupling system (AB ExionLC) and a high-resolution mass spectrometer (AB TripleTOF 6600 plus).

### 2.4. Preparation of BSHXF-Medicated Serum

To prepare serum containing BSHXF, 30 Sprague-Dawley rats (7 weeks, 250 ± 20 g) were supplied from Jinan Pengyue Animal Co. Ltd. (No. 370726211100792337). This research proposal was authorized by the Experimental Animal Ethics Committee of the Affiliated Hospital of Shandong University of Traditional Chinese Medicine (No. 2020-35). The animals were observed for a week in an SPF quarantine room to check for symptoms. Then, all animals were moved to the rat feeding room and provided with enough space, and the standard temperature was controlled (22°C ± 2°C) where they could roam, drink, and eat freely. We divided randomly the rats (*n* = 30) into two groups: the normal group (*n* = 15) and the BSHXF-treated group (*n* = 15). Various concentration of BSHXF was determined by the equivalent dose coefficient conversion method. In the BSHXF-treated group, according to the body weight of rat, each rat was administered BSHXF (final concentration of 2 g/ml) in a volume of 2 ml once per day for 6 weeks. Rats in normal group are gastric with the same volume of saline solution. At 1 h after the last gavage treatment, we anesthetized the rats by injecting pentobarbital sodium. We took blood from the abdominal aorta and left it standing at room temperature for 3 hours. The obtained serum containing BSHXF was filtered with a 0.22 mm strainer and stored at -20°C after static, inactivation, and aseptic filtration (Supplementary material S1).

### 2.5. Extraction and Foster of Human NP Cells

We transferred disc tissue to the laboratory within 60 minutes. After abstersion with phosphate-buffered saline (PBS), the tissue was cut into 1 cm^3^ fragments. The NP cells were obtained from the tissue fragments by digestion with a 0.25% trypsin solution for 30 minutes and 0.2% type II collagenase for 4 h. NP cells were obtained by passage through a 200 *μ*m filter and were scrubbed with PBS two times. Finally, we resuspended the cells with DMEM/F12 containing 15% fetal bovine serum and 100 mg/ml streptomycin, 100 U/ml penicillin, and 1% L-glutamine and placed cells at 37°C in constant temperature incubator with 5% CO_2_. The culture medium was replaced for the first time after 7 days and every 3 days thereafter. When the cells grew to 85%-90% confluence, they were digested with pancreatic enzyme and subcultured in culture dish. Third, passage cells were seeded onto 96-well culture plate.

### 2.6. Identification of NP Cells

NP cells cultured were collected by digesting with EDTA-free trypsin and then centrifuging at 300 × *g* for 5 minutes. After abstersion with PBS, the cells were stained with fluorescent monoclonal CD24 antibodies diluted with 3% BSA. After being incubated away from light for 30 minutes, we added the PBS containing 1% paraformaldehyde to the mixture containing cells.

### 2.7. Assay of Cell Viability

The impact of BSHXF on TNF-*α*-treated NP cells was determined by Cell Counting Kit-8. The cells in a good state were trypsinized and inoculated in experimental plates with 8 × 10^3^ cells. Undergoing the cultivation, the cells were intervened with 100 *μ*l of fresh culture medium containing 10 ng/ml TNF-*α* for another 24 h. Then, we designed a concentration gradient (5%, 10%, 15%, and 20%) of BSHXF-contained serum to intervene cells and detect its optimum concentration. The cell viability was detected using the CCK-8 method in accordance with the CCK-8 kit instructions. First, we added CCK-8 solution to the medium and placed NP cells in 37°C incubator for 4 h. After cultivation, we measured the absorbance (460 nm) of each well and calculated the optimal dose.

### 2.8. Real-Time Quantitative PCR Detection (RT-qPCR)

We extracted total RNA from cells in each group with a SPARK easy Improved Tissue/Cell RNA kit (Spark Jade: AC0202) in the light of the manufacturer's recommended procedures. Briefly, we added lysis solution to the treated NP cells in each groups, which were centrifuged at 13400 × *g* for 60 seconds. Next, an equivalent volume of 70% ethanol was added, and the solution was immediately purified over an RA adsorption column. Afterward, we added 700 *μ*l deproteinized solution RW1 to the RW absorption tube. Then, the procedure which the RW rinsing solution was added and centrifuged was repeated twice. The RA adsorption column was placed back into an empty collecting tube. Finally, total RNA was extracted by adding sterile enzyme-free water to RNA adsorption columns and centrifugation. After the RNA concentration was determined by spectrophotometry, they are used to obtain cDNA with an SPARK script IIRT Plus Kit (Spark Jade: AG0304). The qPCR reaction which was mixed with 2 × SYBR Green qPCR Mix (Spark Jade: AH0104) was set at 94°C for 3 minutes. After the incubation at 94°C for 10 s and 60°C for 30 s. The relative expression levels of target genes and reference genes were quantified by 2^−*ΔΔ*CT^ method. We used glyceraldehyde-3-phosphate dehydrogenase (GAPDH) for the purpose of an internal control. The primers used are listed in Table 2.

### 2.9. Western Blotting

We detected the level of protein by western blotting. After intervened and immediately washed with procooling PBS twice, the cells were lysed on ice with protein lysis buffer containing protease inhibitor (Solarbio) for 30 min, centrifuged at 300 × *g*. The protein solution was extracted. A 15% sodium dodecyl sulfate polyacrylamide gel electrophoresis was performed on 15 micrograms of protein. The process of film transfer is performed on a wet transfer method. The polyvinylidene fluoride (PVDF) membranes of adhesive proteins were incubated in 5% milk. Then, the PVDF membranes were combined with primary antibodies (anti-AMPK (Abcam32047) diluted from 1 : 1000 to 1 : 5000, anti-p-AMPK (Abcam133448), diluted from 1 : 1000 to 1 : 10000, anti-STRT1 (Abcam110304) concentration of 1-0.125 *μ*g/ml, anti-LC3 (catalog number: 14600-1) diluted from 1 : 1000 to 1 : 5000, anti-p62 (Abcam109012) diluted from 1 : 10000 to 1 : 50000, anti-c-caspase3 (Abcam32351) diluted 1 : 5000, anti-GAPDH (Abcam8245) diluted from 1 : 500 to 1 : 10000, anti-cytochrome-c (Abcam13575), concentration of 1-5 *μ*g/ml, anti-beclin-1 (Abcam207612) diluted 1 : 2000, anti-collagen II (Abcam188570), diluted from 1 : 1000 to 1 : 10000, anti-aggrecan (Abcam3778), concentration of 1 *μ*g/ml, anti-ADAMTS4(Abcam84792), diluted from 1 : 200 to 1 : 500, anti-MMP3(Abcam52915), diluted from 1 : 1000 to 1 : 20000 at 4°C overnight. Afterward, the PVDF membranes were washed with Tris-buffered saline containing Tween (TBST) for 10 minutes each time. Then, the secondary antibodies (goat anti-mouse IgG (H+L), horseradish peroxidase (HRP)), was used to combine with the blot for 2 h. The western bright electrochemiluminescence (ECL) was dropped onto PVDF membrane for exposure processing to obtain the image.

### 2.10. Immunofluorescence Staining

The NP cells fixed with 4% paraformaldehyde were washed with PBS, perforated with PBS solution containing 0.3% Triton X-100, and blocked with 5% BSA. The primary antibodies against MMP3 (1 : 500) or collagen II (1 : 1000) were applied to incubate the cells overnight. Then, Alexa Fluor® 488 goat anti-rabbit secondary antibodies (ab150077, diluted from 1 : 200 to 1 : 1000) were added to the cells for incubation for 2 h. And the nuclei was stained with DAPI (ab104139). Fluorescence was measured by fluorescence microscope.

### 2.11. Transmission Electron Microscopy (TEM)

The treated NP cells were fixed in 2.5% glutaraldehyde overnight and 1% osmium tetroxide aqueous solution for 1 h and dark stained in 2% uranyl acetate aqueous solution for 1 h. The cells were dehydrated in graded ethanol for 15 minutes and then infiltrated overnight with epoxy propane embedding medium. Uranyl acetate and lead citrate was applied to poststain obtained ultrathin sections. The slice was observed using TEM.

### 2.12. Cell Transfection

Autophagy flux was assessed using the recombinant adenovirus vector pAV-CMV-TagRFP-SEP-LC3 (S2231, Sunbio Medical Biotech). The cells were inoculated in 6-well plates, and the recombinant adenovirus solution of PDV-CMV-TagRFP-SEP-LC3 with a concentration of 1 × 10 8 PFU/mL was added into the NP cells. The transfection efficiency was determined by fluorescence microscopy. Cells were observed and measured by laser scanning confocal microscopy. The red spots are autophagolysosomes, and the yellow spots are autophagosomes. The strength of autophagy flow can be clearly seen by counting spots of different colors.

### 2.13. siRNA Transfection

siRNA targeting SIRT1 is used for SIRT1 knockout. Human SIRT1 siRNA and negative control siRNA (NC-siRNA) were designed and synthesized by GenePharma. NP cells were cultured in 6-well plates. When the cells have reached 70% fusion, the NP cells are carefully transfected with specific siRNA as the manufacturer's instructions. After transfection, cells were collected for subsequent experiments.

### 2.14. MitoTracker Assay

We applied MitoTracker staining to detect mitochondria in each group of NP cells with the MitoTracker assay kit (C1049; MitoTracker Red CMXRos, Beyotime Biotechnology). The procedure was carried out according to the instructions. In short, after intervention of the NP cells, the culture medium was replaced, and the prepared MitoTracker Red CMXRos working solution was added and fostered for 30 minutes. After the MitoTracker working solution was discarded, the cells were replaced with fresh culture medium and further cultured at 37°C.

### 2.15. Measurement of Superoxide Dismutase (SOD) Activity and ATP Levels

SOD enzymatic activity and ATP levels in NP cells were detected using a Total Superoxide Dismutase (T-SOD) Colorimetric Assay Kit (WST-1 method) (Elabscience: E-BC-K020-M) and Adenosine Triphosphate (ATP) Colorimetric Assay Kit (E-BC-K157-M), respectively, in the light of the manufacturer's instruction. Human NP cells were fostered at 37°C in 5% CO_2_ after being treated. Briefly, the cells were scraped off with a scraper and collected under the centrifugation at 1000 × *g* at 4°C for 10 minutes. Then, the 10^6^ cells were lysed with 300 *μ*l-500 *μ*l of homogenate medium and centrifuged. The supernate was collected and measured by adding the relevant test working fluid.

### 2.16. Detection of ROS Level

After the cells were treated, we measured the level of ROS with a ROS Assay Kit (Beyotime, SOO33S). Briefly, after treatment as instructions, the cells were incubated by 10 *μ*M 2′-7′-dichlorodihydrofluorescein diacetate (DCFH-DA) for 25 minutes. The intracellular ROS fluorescence intensity was observed using laser confocal microscope.

### 2.17. TUNEL Staining

NP cells were analyzed by a TUNEL Apoptosis Assay Kit (Beyotime, C1086) in the light of the kit instructions. The cells in 12-well plates were performed to fixation with 4% paraformaldehyde, permeabilized treatment with prepared PBS containing 0.3% Triton X-100. Then, we added TUNEL detection solution to the cells and cultured at the 37°C in the dark for 60 minutes. In the end, the cells were observed using a confocal microscope.

### 2.18. Flow Cytometry for Apoptosis Analysis

We determined the apoptosis percentage of NP cells using an Annexin V-FITC/PI Apoptosis Detection kit (Yeasen, 40302ES20), and the procedure was carried out in the light of the kit instructions. After the digestion of 0.25% pancreatic enzyme (without EDTA) and centrifugation of 300 × *g*, the cells were collected. Then, the collected NP cells were performed to abstersion with PBS and centrifugation for 5 minutes. After 100 *μ*l of 1 × binding buffer was added, the cells were fostered with 5 *μ*l of Annexin V-FITC and 10 *μ*l of PI staining solution in dark place for 15 minutes. Finally, the cells were mixed with 400 *μ*l of 1 × binding buffer and quantified by flow cytometry.

### 2.19. Statistical Analysis

All experiments were repeated independently at least three times, and the experimental data were expressed as mean ± SD. One-way analysis of variance (ANOVA) was adopted to analyze significant differences. *P* < 0.05 indicated statistical significance.

## 3. Results

### 3.1. Impact of BSHXF on NP Cell Viability

**Figure 1:**
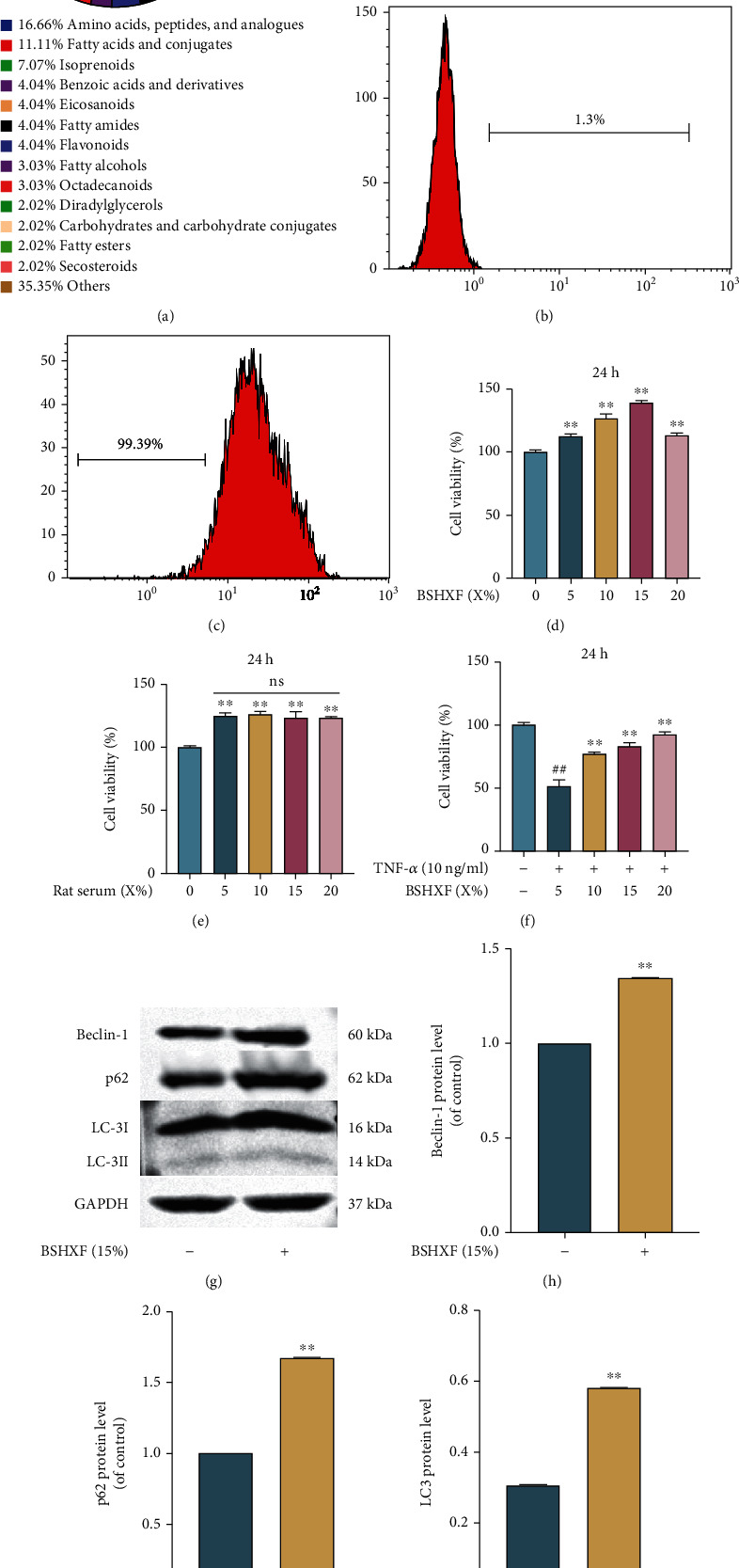
Positive effect of BSHXF on the viability of NP cells. (a) The identification about BSHXF components. (b–c) The identification of NP cells with CD24. (d) The impact of the BSHXF (5%, 10%, 15%, 20%, and 25%) on the NP cells was detected by a CCK-8 assay. (e) The proliferation of NP cells treated with various concentrations of serum without BSHXF was measured by CCK-8 assay. (f) The effect of BSHXF was detected using the CCK-8 assay after the cells were induced by TNF-*α*. (g–j) Western blotting was applied to determine the positive effect of BSHXF on NP cells. The group treated with serum with or without BSHXF; ^∗^*P* < 0.05 and ^∗∗^*P* < 0.01; the group treated with TNF-*α*, ^#^*P* < 0.05 and ^##^*P* < 0.01.

The procedure to prepare serum containing BSHXF and its components is shown in Figure 1(a). NP cells were identified by flow cytometry (Figures 1(b) and 1(c)). To determine the optimal density of BSHXF, we fostered the cells with diverse concentrations of serum containing BSHXF. Then, different concentrations of serum without BSHXF was added to the cells to detect whether serum alone enhances the proliferation of NP cells. After 24 h of treatment, the CCK-8 assay results indicated that the appropriate concentration was 15% (Figures 1(d) and 1(e)). After the cells were intervened with TNF-*α* (10 ng/ml), different concentrations of serum containing BSHXF were added. As shown in Figure 1(f), the optimal concentration was obtained with 15%. Moreover, the positive effect on NP cells was detected by western blotting (Figures 1(g)–1(j)).

### 3.2. Impact of BSHXF on Matrix Degradation Induced by TNF-*α*

**Figure 2:**
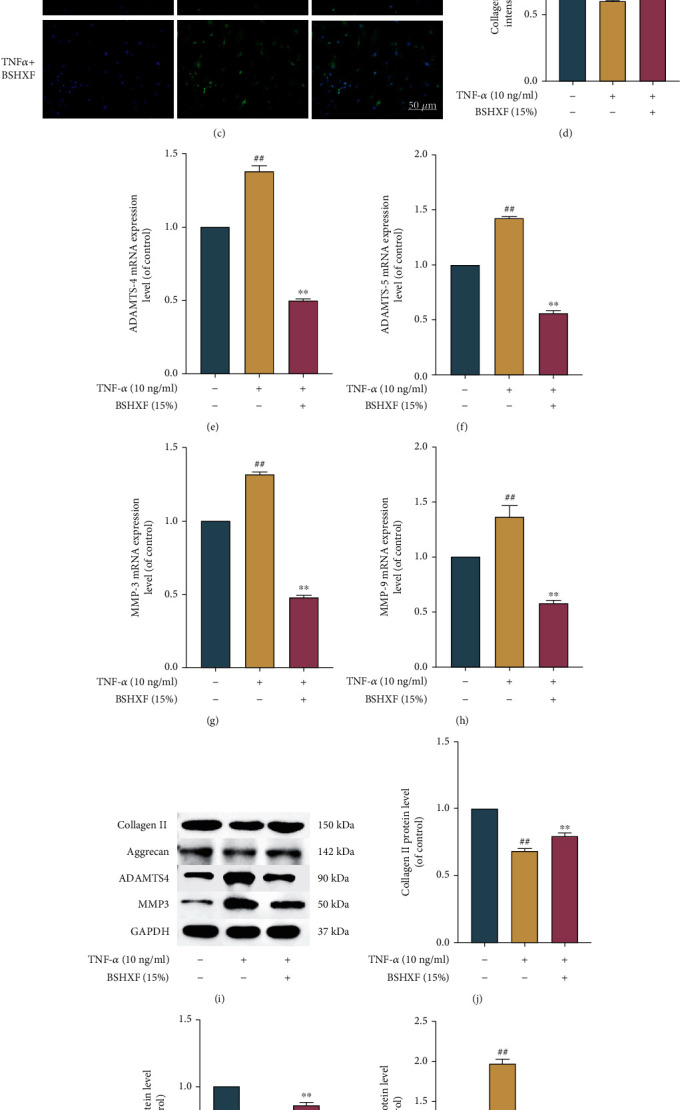
BSHXF treatment alleviates ECM induced by TNF-*α*. (a–d) Immunofluorescence staining of MMP-3 and collagen II in BSHXF-treated NP cells induced by TNF-*α*. (e–h) The mRNA expression levels of MMP-3, MMP-9, ADAMTS-4, and ADAMTS-5 were determined by RT-PCR. (i–m) The protein level of collagen II, aggrecan, ADAMTS-4, and MMP3. The group treated with serum containing BSHXF, ^∗^*P* < 0.05 and ^∗∗^*P* < 0.01; the TNF-*α* group, ^#^*P* < 0.05 and ^##^*P* < 0.01.

The main characteristics which are considered to be IVDD are as follows: unbalance of ECM synthesis and decomposition in cells and the resulting ECM degradation. The expression of ECM metabolism markers was detected by RT-qPCR, immunofluorescence, and western blotting. The immunofluorescence staining data are as follows. The level of collagen II in the BSHXF-treated cell was more excessive than that in the TNF-*α*-treated cell, but the level of MMP3 in the TNF-*α*-treated cell was more excessive than that in the BSHXF-treated cell (Figures 2(a)–2(d)) Furthermore, after comparison with the control, TNF-*α* upregulated the mRNA level of ADAMTS-4, ADAMTS-5, MMP-3, and MMP-9 and the protein level of ADAMTS-4 and MMP-3. After treatment, BSHXF downregulated the mRNA levels of ADAMTS-4, ADAMTS-5, MMP-3, and MMP-9 (Figures 2(e)–2(h)) and the protein level of ADAMTS-4 and MMP-3 (Figures 2(i)–2(m)).

### 3.3. Impact of BSHXF on Apoptosis, ROS Production, and Damaged Mitochondrial Intervened by TNF-*α*

**Figure 3:**
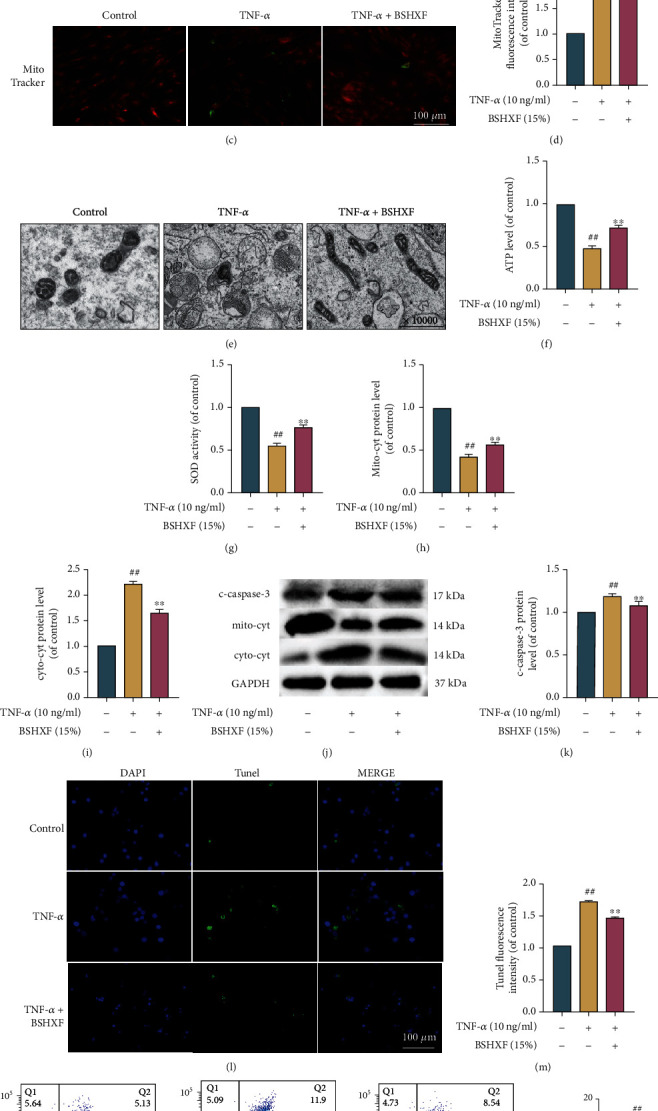
BSHXF treatment antagonizes cell apoptosis, decreases ROS production, and alleviates mitochondrial dysfunction induced by TNF-*α*. (a–b) Typical images and expression of ROS levels. The phalloidin (green) was used to counterstain the cells. Scale bar: 100 *μ*m. (c–e) Typical fluorescence images of NP cells mitochondria. Phalloidin (green) was used to counterstain cells. Scale bar: 100 *μ*m. (f–g) The determination of the ATP and SOD levels. (h–k) Western blotting was applied to detect the expression of c-caspase-3, mito-cyt, and cyto-cyt.(l and m) The NP cell image of TUNEL staining in each group. The DAPI was used to stain nuclei. Scale bar: 100 *μ*m. (n) Measurement of NP cell apoptosis by flow cytometry. The group treated with serum containing BSHXF, ^∗^*P* < 0.05 and ^∗∗^*P* < 0.01; the TNF-*α* group, ^#^*P* < 0.05 and ^##^*P* < 0.01.

We measured the impact of BSHXF on NP cell apoptosis, ROS production, and mitochondrial function in cells. The ROS kit was used to measure ROS level. The analysis demonstrated that TNF-*α* markedly increased the ROS accumulation, while ROS levels were downregulated by BSHXF (Figures 2(b) and 3(a)). A MitoTracker Test Kit and TEM were applied to examine mitochondrial morphology, and the results showed that serum containing BSHXF reversed TNF-*α*-induced mitochondrial fission (Figures 3(c)–3(e)). ATP and SOD levels in TNF-*α*-induced cells were increased dramatically. After treated by BSHXF, the ATP and SOD levels were increased (Figures 3(f) and 3(g)). In addition, the mitochondrial apoptotic pathway-associated proteins cleaved caspase-3 and cytochrome c in cells were measured. It was reported that oxidative stress-induced apoptosis was activated at least through mitochondrial pathway. Western blotting analysis indicated that TNF-*α* upregulates the expression of caspase-3 and cytoplasmic cytochrome c in cells and downregulates the expression of mitochondrial cytochrome c. When the cells were treated with BSHXF, the expression of caspase-3 and cytoplasmic cytochrome c was downregulated, and the expression of mitochondrial cytochrome c was upregulated (Figures 3(h)–3(k)). Additionally, the TUNEL staining results demonstrated that, BSHXF withstood TNF-*α*-treated apoptosis in NP cells (Figures 3(l) and 3(m)). Finally, BSHXF retarded the apoptosis intervened by TNF-*α* in cells (Figure 3(n)).

### 3.4. Impact of BSHXF on Autophagy in NP Cells

**Figure 4:**
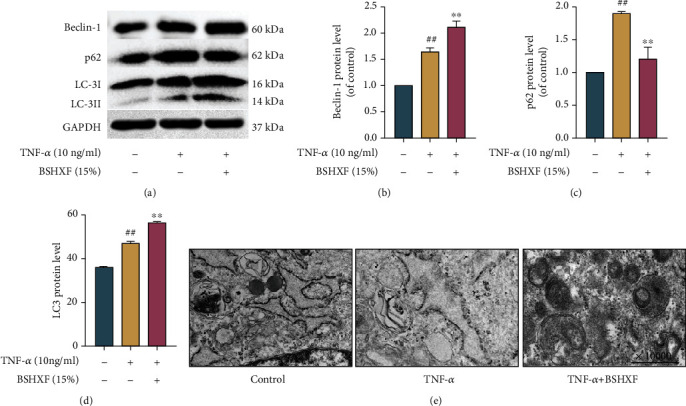
BSHXF reinforces the effect of autophagy in NP cells. (a–d) Western blotting was applied to measure the level of LC3, beclin-1, and p62. NP cells were intervened in the presence of TNF-*α* and BSHXF. (e) Electron microscopy images of autophagosomes. NP cells were fostered with TNF-*α* and BSHXF. The group treated with serum containing BSHXF, ^∗^*P* < 0.05 and ^∗∗^*P* < 0.01; the TNF-*α* group, ^#^*P* < 0.05 and ^##^*P* < 0.01.

Damaged mitochondria are degraded by autophagolysosomes and release energy, which provides energy for new mitochondria and restores mitochondrial function. The western blotting showed that BSHXF upregulated the level of LC-3II/LC-3I and beclin-1. Moreover, p62 can reflect the state of autophagic flow because it was a specific substrate degraded in autophagic lysosomes. Western blotting analysis showed that BSHXF upregulated the expression of beclin-1, and the ratio of LC-3II/LC-3I showed the same upregulation. The results demonstrated that the level of p62 was upregulated in TNF-*α*-induced group and that the level was decreased by BSHXF treatment (Figures 4(a)–4(d)). Finally, autophagosomes and autophagolysosomes were observed by TEM. Comparison with that in the TNF-*α*-induced NP cells, the quantity of autophagosomes and autophagolysosomes in the BSHXF-treated NP cells rose (Figure 4(e)). Collectively, the refine result indicated that BSHXF upregulated autophagic flux and enhanced the effect of autophagy.

### 3.5. Regulation of BSHXF on Autophagic Flux

**Figure 5:**
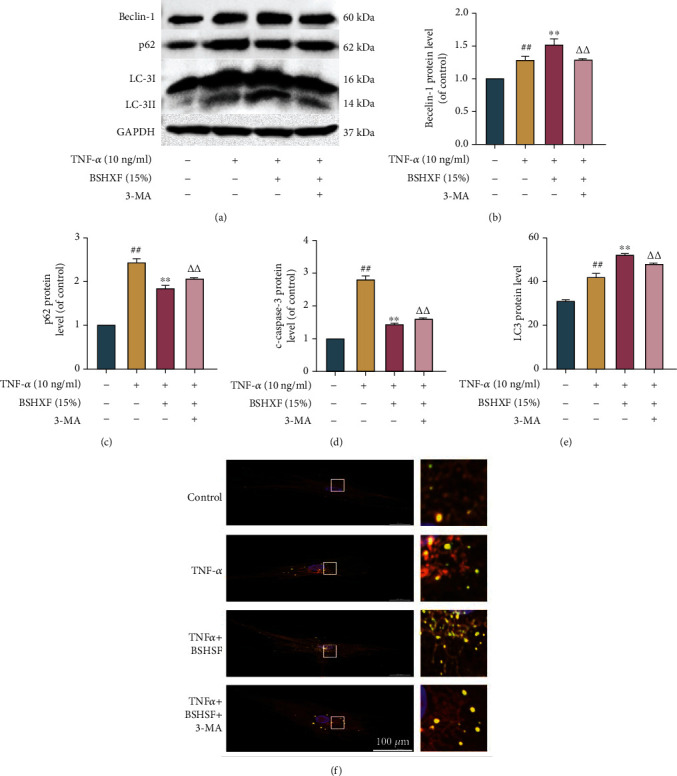
BSHXF upregulates autophagic flux in NP cells. (a–e) Western blotting was applied to determine the expression of LC3, beclin-1, p62, and c-caspase-3. The cells were intervened in the presence of TNF-*α* and 3-MA with or without BSHXF. (f) Changes in autophagy flux were detected by fluorescence microscopy (yellow dots represent autophagosomes and red dots represent autophagolysosomes). The group with serum containing BSHXF, ^∗^*P* < 0.05 and ^∗∗^*P* < 0.01; the TNF-*α* group, ^#^*P* < 0.05 and ^##^*P* < 0.01; and the 3-MA-treated group, ^∆^*P* < 0.05 and ^∆∆^*P* < 0.01.

We intervened NP cells with 3-MA, an autophagy blocker, for 24 h, and exposed them to BSHXF and TNF-*α* to investigate whether BSHXF had a protective effect by increasing autophagy flux. According to the western blotting results, 3-MA decreased the ratio of LC-3II/LC-3I and the level of p62 in cells treated with TNF-*α* and BSHXF, which showed autophagy inhibition. Next, we assessed the apoptosis-related markers and found that 3-MA eliminated the BSHXF-induced decrease of proapoptotic caspase-3. The results showed that the prophylaxis function of BSHXF on apoptosis induced by TNF-*α* was weakened by 3-MA. Furthermore, oxidative stress and mitochondrial impairment induced by TNF-*α* were improved by the treatment of BSHXF in NP cells, which showed decrease in ROS accumulation and increases in SOD and ATP levels. However, 3-MA reversed BSHXF-mediated amelioration. Overall, these results demonstrated that apoptosis and decrease in autophagy flux induced by TNF-*α* were inhibited by BSHXF (Figures 5(a)–5(e)).

### 3.6. Impact of the AMPK/SIRT1 Pathway on Upregulation of Autophagic Flux Mediated by BSHXF

In order to determine whether blocking AMPK activation would mitigate NP degeneration induced by TNF-*α*, 10 ng/ml TNF-*α* was used to stimulate the cells with or without the AMPK inhibitor dorsomorphin. The results of western blotting showed that BSHXF treatment regulated autophagic flux under the activation of AMPK. Moreover, after SIRT1 was knocked out, the NP cells were treated with 10 ng/ml TNF-*α*, serum containing BSHXF and AMPK inhibitors dorsomorphin, respectively. The final results showed that in human NP cells, dorsomorphin inhibited the BSHXF-mediated upregulation of AMPK phosphorylation and SIRT1 expression.

**Figure 6:**
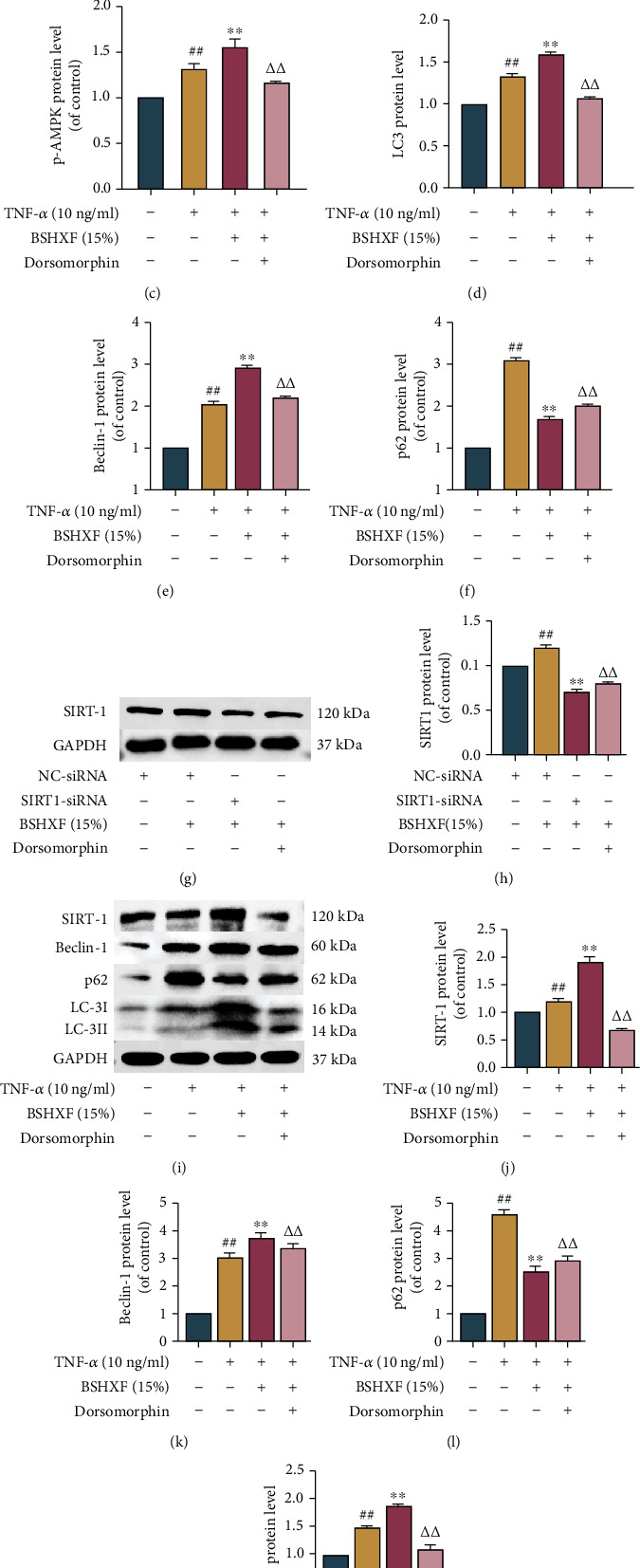
The AMPK/SIRT1 pathway engages in autophagy activation. (a–m) The indicators of AMPK, p-AMPK, and SIRT-1 were measured by western blotting. The dorsomorphin was used to intervene with the cells fostered with TNF-*α* and BSHXF. The group treated with serum containing BSHXF, ^∗^*P* < 0.05 and ^∗∗^*P* < 0.01; the TNF-*α* group, ^#^*P* < 0.05 and ^##^*P* < 0.01; the dorsomorphin-treated group, ^∆^*P* < 0.05 and ^∆∆^*P* < 0.01.

These results demonstrated that activation of autophagy mediated by BSHXF was associated with the AMPK/SIRT1 pathway (Figures 6(a)–6(m)).

## 4. Discussion

In our theory of Traditional Chinese medicine, the common pathogenic factors of IVDD are formed by kidney weakness and blood stasis. On the one hand, blood stasis refers to the inability of qi and blood to function due to external factors. Its occurrence involves susceptibility to “wind pathogens,” “dampness pathogens,” and “cold pathogens” and is associated with kidney function. On the other hand, kidney vacuities also cause degeneration of the IVDD. Therefore, TCM mainly concentrates on tonifying the kidney and promoting blood circulation.

TCM has been applied to treat various diseases, respectively, in medicine, surgery, gynecology, and pediatrics, in many countries, such as China and Japan. Single Chinese herbal medicine or Chinese herbal formula is not only effective in the clinic and cheap on the cost but also has few side effects. BSHXF, which consists of five TCM herbs and is used in the treatment of kidney vacuities [55]. In addition, it was reported that some compounds in BSHXF play important roles in orthopedic diseases. Naringin mitigates on osteoporosis by enhancing the proliferation of bone marrow stromal cells (BMSCs) and promoting their osteogenic differentiation [56]. The analysis showed that BSHXF regulates the AMPK/SIRT1 pathway and autophagy in NP cells, which restrains TNF-*α*-induced apoptosis and matrix degeneration and exerts antiapoptosis and antioxidant effects. The major findings provide novel insights into the molecular mechanisms by which BSHXF affects IVDD and has a significant impact on validating the clinical use of BSHXF to treat LBP caused by IVDD in humans. Elevated levels of inflammatory factors lead to excessive inflammatory responses of IVD cells, which in turn lead to oxidative damage and ECM degradation, which are deemed to be the major pathological factors of IVDD [57]. In addition, the increased ROS levels lead to mitochondrial dysfunction and the release of cytochrome c, which results in NP cell apoptosis [58, 59]. Therefore, this is a very effective mechanism to inhibit the proinflammatory factors [60] and ECM degeneration and restore autophagic flux.

Autophagy can selectively remove dysfunctional and damaged organelles or proteins to maintain normal cell metabolism and function, which is regarded as a regulatory mechanism of cell homeostasis [61]. Macroautophagy is the best-characterized form of autophagy, which is accompanied by the formation of autophagosomes. Autophagosomes surround part of the cytoplasm and ultimately fuse with lysosomes [62, 63]. Moreover, the protective effect of autophagy on NP cells is mainly reflected in cell growth, survival, proliferation, and differentiation [64, 65].

Our experimental result showed that inflammatory factors, including TNF-*α*, not only increased the level of ROS and mitochondrial dysfunction but also led to cell apoptosis. TNF-*α* increases the level of MMPs in NP cells. Furthermore, TNF-*α* induces the highest MMP-3 level compared to other MMPs [66]. The results demonstrated an upregulation in MMP3 expression and a downregulation in collagen II expression in the TNF-*α*-induced cells. ROS upregulated the level of caspase-3 in cells, accelerated the release of cytochrome c from mitochondria, and exacerbated the rate of apoptosis. Related studies have manifested that the expression of matrix degrading protease can also be caused by the increase of intracellular ROS level [67]. The levels of ATP and SOD were increased in the TNF-*α* group, which resulted in cell apoptosis and ECM degradation. Excessive apoptosis reduces the number of NP cells. Here, we showed that apoptosis and ECM degradation were closely related to physiological and pathological mechanisms of IVDD and played an important role in IVDD. When NP cells were induced with TNF-*α*, which can promote ROS accumulation, the mitochondrial dysfunction and abnormal structures were observed by confocal microscopy. Moreover, three cellular apoptosis-related proteins (caspase-3, mito-cyt, and cyto-cyt) were assessed, and the results demonstrated that TNF-*α* could activate these proteins and destabilize mitochondrial function and structure. Thus, these data showed that TNF-*α* upregulated ROS, thereby activating apoptosis signaling molecules and resulting in ECM degradation.

In our study, we found a risen ratio of LC3II/I and level of beclin-1 in BSHXF-treated cells. Our results also showed that BSHXF could increase autophagic flux and upregulate autophagic activity. The RT-qPCR and western blotting data revealed that BSHXF could promote the synthesis of collagen II and aggrecan and reduce the level of MMP-3 and ADAMTS-4. Therefore, inhibiting ECM degradation effectively slows down IVDD. The results showed that apoptosis factor activity could be suppressed. When the 3-MA was applied, the effect of BSHXF was abrogated to a large extent. When autophagic flux was diminished in cells, the rates of apoptosis and ECM degradation were increased. The results showed that BSHXF could delay degeneration of the IVD by increasing autophagic flux.

As a master sensor of energy, AMPK plays pivotal roles in regulating energy levels to suit cellular homeostasis [68]. Moreover, the crucial role of SIRT1 in cell energy balance is mainly reflected in the quality of mitochondria and the safety of normal mitochondrial function [69, 70]. AMPK/SIRT1 is a crucial pathway that regulates energy. Under normal conditions, AMPK binds to SIRT1 and keeps SIRT1 in an inactive state. The increase in AMPK activity promotes an increase in autophagy, and SIRT1 activation increases mitochondrial biogenesis. SIRT1 is activated under high ROS conditions [71]. Multiple factors regulate AMPK/SIRT1, including inflammatory factors, nutritional deficiency, and oxidative damage, and are associated with IVDD [72]. When the AMPK/SIRT1 signaling was activated, the level of p-AMPK and SIRT1 in the BSHXF-treated group rose in comparison with that in the TNF-*α*-induced group. When the AMPK inhibitor dorsomorphin was added, the expression of both factors was inhibited. Therefore, we concluded that BSHXF could promote ECM synthesis and restrain NP cell apoptosis by activating AMPK/SIRT1 (Supplementary material S2).

## 5. Conclusions

The study shows that BSHXF treatment regulates oxidative stress and restores mitochondrial function to weaken cell apoptosis and increases ECM synthesis through AMPK/SIRT1. The activation of AMPK/SIRT1 protects cells against inflammatory reactions and oxidative stress that causes apoptosis and ECM degradation. Moreover, excessive oxidative stress upregulates autophagy, and BSHXF regulates autophagy in response to AMPK/SIRT1 signaling in IVDD.

## Figures and Tables

**Table 1:** Components of Bushen Huoxue Formula.

Botanical name	Chinese name	Origin (China)	Plant part	Dosage (g)	No.
Aconitum carmichaelii Debx	Fuzi	Sichuan	Root	10	50042970
Rehmannia glutinosa Libosch	Dihuang	Liaoning	Root	20	29208879
Morinda officinalis	BaJitian	Guangdong	Root	15	129889
Salvia miltiorrhiza Bunge	Xianmao	Zhejiang	Root	10	303703
Curculigo orchioides	Danshen	Shandong	Root	20	183206

**Table 2:** Primers used in qRT-PCR.

Name	Primer	Sequence
MMP-3	Forward	GGGTCTCTTTCACTCAGCCAACAC
	Reverse	ACAGGCGGAACCGAGTCAGG
MMP-9	Forward	CGTCTTCCAGTACCGAGAGAAAGC
	Reverse	CTTGGTCCACCTGGTTCAACTCAC
ADAMTS-4	Forward	AGAGTCCTGCCAGCGGTCAAG
	Reverse	TCTGCCACCACCAGTGTCTCC
ADAMTS-5	Forward	TTGTGAGTGAGACTGTGCGG
	Reverse	AACCTGCCTATGTTCCCTGC
GAPDH	Forward	TCAAGAAGGTGGTGAAGCAGG
	Reverse	TCAAAGGTGGAGGAGTGGGT

## Data Availability

The research data used to support the findings of this study are included within the article. The research data used to support the findings of this study are included within the supplementary information file(s).

## Abbreviations

LBP: Low back pain
IVDD: Intervertebral disc degeneration
ECM: Extracellular matrix
NP: Nucleus pulposus
ROS: Reactive oxygen species
TNF-*α*: Tumor necrosis factor-*α*
AF: Annulus fibrosus
CEP: Cartilage endplate
IL-6: Interleukin-6
MMPs: Matrix metalloproteinases
ADAMTS: Thrombospondin motifs
ATP: Adenosine triphosphate
AMP: Adenosine monophosphate
ADP: Adenosine diphosphate.

## Supplementary Materials

Supplementary Materials Supplementary materials 1: the SD rats were given the decoction of Bushen Huoxue Formula, and then, the serum containing Bushen Huoxue Formula was prepared through the abdominal aorta. Supplementary materials 2: diagram of the mechanism pathway of tumor necrosis factor-*α* acting on cells, causing inflammatory response and mitochondrial somatic dysfunction, leading to nucleus pulposus cell apoptosis. Supplementary materials 3: graphical abstract of cell apoptosis caused by the imbalance between autophagy and apoptosis and the intervention of Bushen Huoxue Formula.
