# Supplementary material for: Bushen Huoxue Formula Modulates Autophagic Flux and Inhibits Apoptosis to Protect Nucleus Pulposus Cells by Restoring the AMPK/SIRT1 Pathway
Source: Biomed Res Int. 2022 May 27;2022:8929448. doi: 10.1155/2022/8929448 (PMC9167005; doi:10.1155/2022/8929448)
Supplement: Supplementary Materials — 1: the SD rats were given the decoction of Bushen Huoxue Formula, and then, the serum containing Bushen Huoxue Formula was prepared through the abdominal aorta. Supplementary materials 2: diagram of the mechanism pathway of tumor necrosis factor-α acting on cells, causing inflammatory response and mitochondrial somatic dysfunction, leading to nucleus pulposus cell apoptosis. Supplementary materials 3: graphical abstract of cell apoptosis caused by the imbalance between autophagy and apoptosis and the intervention of Bushen Huoxue Formula. [file 8929448.f1.docx]

**Supplementary material**

**S1.Preparation of serum-containing BSHXF** BSHXF, which is composed of five kinds of herbal medicine (Aconitum carmichaeli Debx, Rehmannia glutinosa Libosch, Morinda officinalis, Salvia miltiorrhiza Bunge and Curculigo orchioides Gaertn) was decocted before gavaged to Rat. Then, we took blood from the abdominal aorta and left it standing at room temperature for 3 hours. The obtained serum containing BSHXF was filtered with a 0.22-mm strainer and stored at -20 **℃.
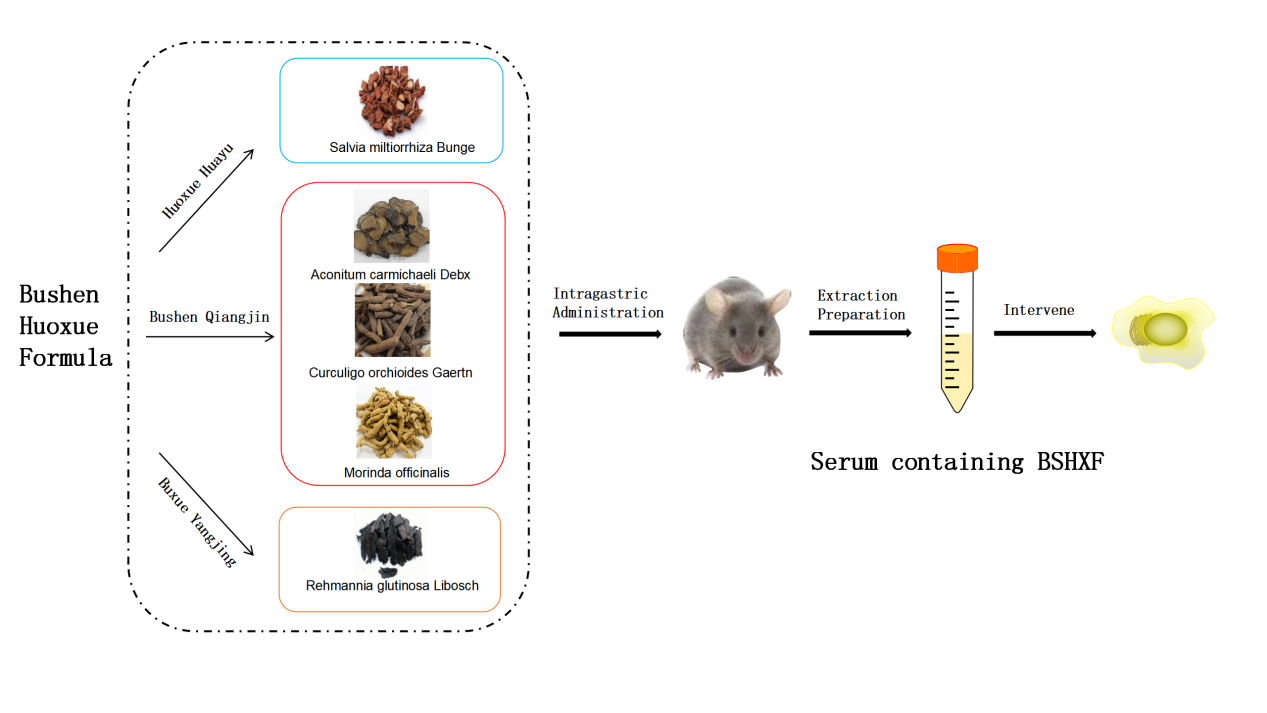
**

**S2.The mechanism of BSHXF on NP cells** Tumor necrosis factor-α stimulates caspase-8 secretion of nucleus pulposus cells, and causes mitochondrial dysfunction, resulting in cytochrome C secretion from mitochondria to cytoplasm. Cytochrome C stimulates caspase-9 secretion, and together with caspase-8 stimulates caspase-3 activation, leading to nucleus pulposus cell apoptosis and extracellular matrix degradation. AMPK and SIRT1 regulate energy homeostasis, reduce ROS accumulation, and maintain normal mitochondrial function

**
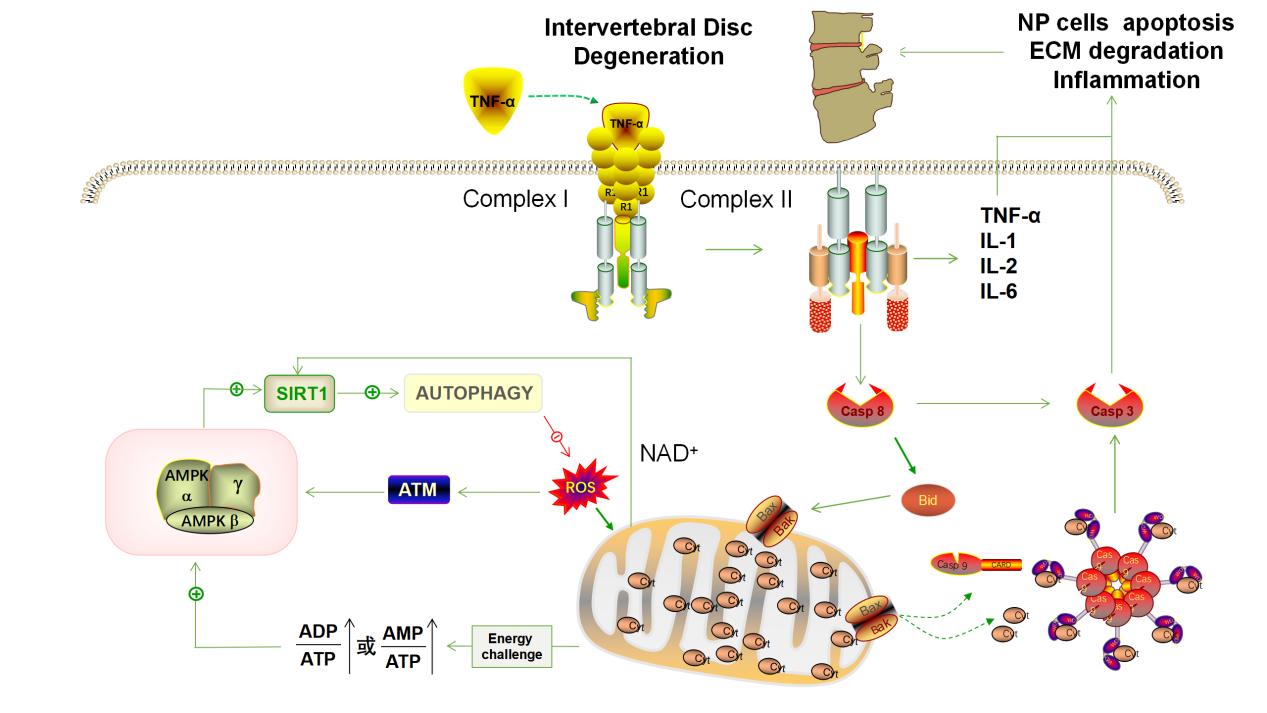
**

# **S3 Graphical abstract** Autophagy and apoptosis are two important processes in intervertebral disc degeneration, and the imbalance between them leads to intervertebral disc degeneration. Apoptosis of nucleus pulposus cells is mainly caused by the release of cytochrome C in mitochondria. This study explored whether Traditional Chinese medicine can regulate autophagy, enhance autophagy flux, and restore the balance between autophagy and apoptosis

**
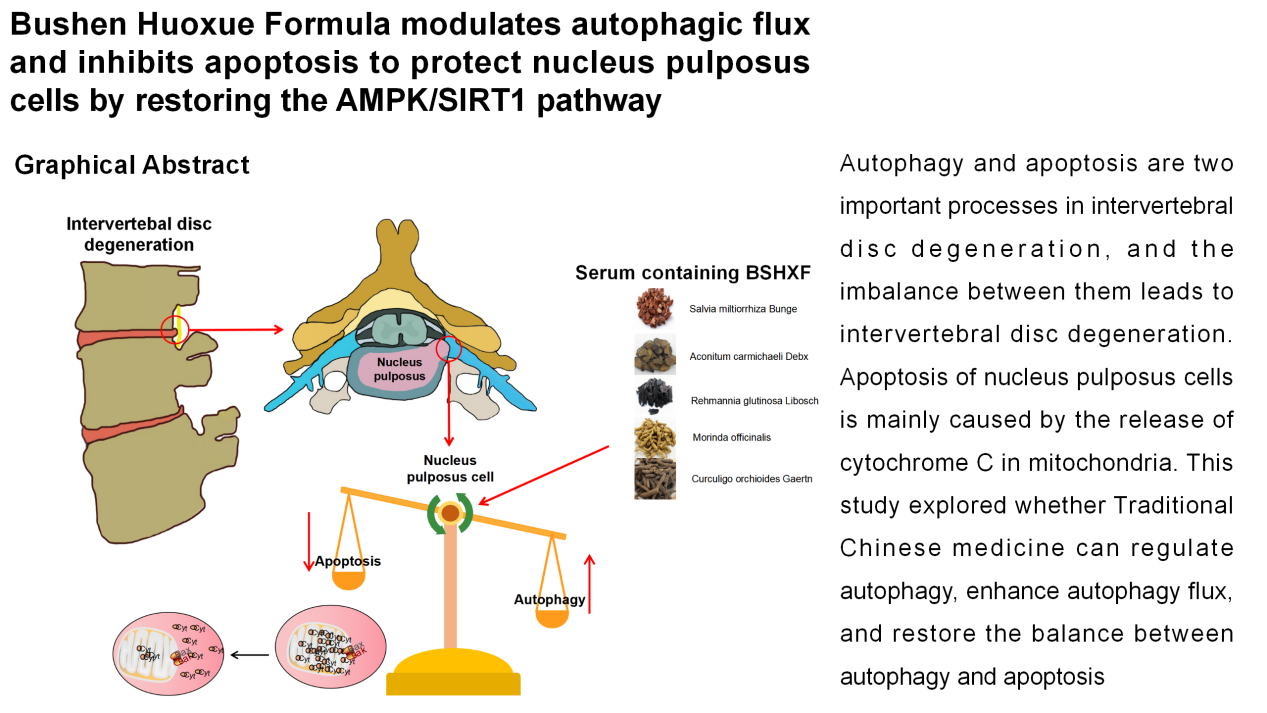
**
